# Lactate Clearance of the Adsorber Cytosorb^®^ in Critically Ill Patients: A Post-Hoc Analysis of the Cyto-SOLVE Trial

**DOI:** 10.3390/biomedicines13020418

**Published:** 2025-02-10

**Authors:** Vassilissa Wustrow, Caroline Gräfe, Helen Graf, Patrick Scheiermann, Michael Paal, Michael Vogeser, Uwe Liebchen, Christina Scharf

**Affiliations:** 1 Department of Anaesthesiology, LMU University Hospital, LMU Munich, 81377 Munich, Germany; vassilissa.wustrow@med.uni-muenchen.de (V.W.);; 2 Institute of Laboratory Medicine, LMU University Hospital, LMU Munich, 81377 Munich, Germany; michael.paal@med.uni-muenchen.de (M.P.);

**Keywords:** Cytosorb^®^, lactate clearance, blood purification, vasopressors, acute kidney injury, hemodynamics

## Abstract

**Background/Objectives**: Patients with shock suffer from hyperlactatemia, which can lead to endothelial dysfunction. The use of the adsorber Cytosorb^®^ (CS) is recommended in these patients as it may contribute to higher lactate clearance and hemodynamic stabilization. However, it is unclear whether CS can directly adsorb lactate and can therefore increase lactate clearance. **Methods**: The Cyto-SOLVE trial included patients undergoing continuous kidney replacement therapy combined with CS application. Patients with a lactate concentration > 2 mmol/L and measurements of lactate pre- and post-adsorber, as well as measurements in the blood 10 min and 1, 3, 6, and 12 h after initiation were selected. Lactate clearance was calculated using the following formula: bloodflow_(mL/min)_ × concentration_pre−post_/concentration_pre_. A *t*-test was used with the collected samples. Changes in the lactate concentration and vasopressor requirement were recorded before initiation and at the end of therapy. **Results**: Sixty-five lactate concentrations were measured pre- and post-CS application, as well as in patients’ blood, in a total of 14 patients (median age of 52 years, 10 males, median SAPS-II 67). There was no significant change in the lactate concentration pre- and post-CS application (mean pre-CS: 6.7 mmol/L, mean post-CS: 6.9 mmol/L, RR: −0.2, 95% confidence interval (CI): −0.4–0.1, *p* = 0.13, Cohen’s d: 0.90). The mean lactate clearance was −6 mL/min (standard deviation (SD): 21 mL/min), with no correlation with the initial lactate concentration or blood flow. In contrast, the mean lactate clearance measured using the dialyzer was 39 mL/min (SD: 28 mL/min). When comparing values before and after treatment, no significant change was observed in the lactate blood concentrations (mean of 9.0 vs. 8.5 mmol/L), nor in the requirement for vasopressin (median of 1.9 vs. 1.8 IE/h) or norepinephrine (mean of 2.7 vs. 2.6 mg/h). **Conclusions**: The adsorber CS cannot directly adsorb lactate, unlike kidney replacement therapy. Therefore, it is not suitable for achieving faster extracorporeal lactate elimination. Understanding the adsorption spectrum is of great relevance and should be considered when using CS in clinical practice.

## 1. Introduction

The clinical appearance of “shock” is defined as a life-threatening acute circulatory failure associated with inadequate oxygen utilization by the cells, leading to insufficient tissue perfusion and a mismatch between oxygen delivery and oxygen consumption [1]. Several critical acute illnesses, such as septic shock [2], cardiogenic shock [3], acute liver failure, and rhabdomyolysis, can be associated with circulatory insufficiency through micro- and macrocirculatory disturbances, potentially causing organ dysfunction, which may lead to multi-organ failure [4]. Lactate is considered a surrogate marker for anaerobic metabolism, released in response to an oxygen deficit. Elevated concentrations lead to vasodilation, which increases local blood flow to ensure adequate oxygen supply [5]. This exacerbates circulatory insufficiency, further worsened by lactic acidosis and reduced catecholamine effectiveness.

Several studies have shown that hyperlactatemia is associated with poor clinical outcomes, as critically ill patients, in particular, experience increased lactate concentrations [6,7,8]. Furthermore, lactate causes endothelial barrier dysfunction [9]. The focus for these patients is the causal therapy of the underlying disease. For example, patients with septic shock require anti-infective drugs to interrupt cytokine release, while patients with compartment syndrome need surgical fasciotomy to halt the release of myoglobin in cases with rhabdomyolysis [10,11]. In addition, symptomatic therapy (e.g., fluid administration and the application of vasopressors or hydrocortisone) is recommended to improve tissue oxygenation and provide endothelial protection [12,13].

Acute kidney injury is common in patients with shock. In addition to oliguria or anuria, the indication for dialysis is often driven by lactic acidosis. Regularly used dialysis membranes achieve high lactate clearance by removing it from the bloodstream through the semi-permeable membrane, resulting in decreasing lactate levels [14,15]. In recent years, the adjunctive use of extracorporeal adsorbers, particularly the adsorber Cytosorb^®^ (CS), has gained importance in the context of shock, promising enhanced lactate clearance by directly adsorbing it from the blood.

CS contains cross-linked small polymer beads, resulting in a large surface area of up to 45,000 square meters. The intention is to adsorb small- and mid-sized hydrophobic molecules with a molecular size of approximately 5–60 kDa [16]. However, the actual adsorption spectrum of this unselective device is not sufficiently described. It can be used in patients with hyperinflammation, rhabdomyolysis, or liver failure. Moreover, it is approved for the removal of ticagrelor and rivaroxaban [17]. It can be used as a stand-alone device, but it is mostly integrated into an extracorporeal circulation system, such as intermittent hemodialysis, continuous kidney replacement therapy (CKRT), and extracorporeal membrane oxygenation (ECMO).

Several authors have reported a significant decrease in lactate blood levels in patients with shock of various origins during CS application. Recently, Hakemi et al. reported a significant decrease in lactate levels in patients with septic shock when combing CKRT and CS [18]. Similar data were observed in a multicenter prospective trial [19]. Friesecke et al. also reported higher lactate clearance in patients with septic shock, although their study lacked a control group [20]. A significant decrease in lactate was also observed in 40 patients with different types of shock following CS application, leading the authors to conclude that the device could effectively reduce lactate concentrations [21].

However, these data do not clarify whether CS directly adsorbs lactate or contributes to shock reversal or whether other interventions are responsible for the decrease in lactate concentration. Since the adsorber is commonly combined with CKRT, and it is known that lactate is removed by dialysis, it would be beneficial to consider the extracorporeal lactate clearance of dialysis and the adsorber separately, which has not been carried out in a study so far.

To date, the clearance of the adsorber CS, particularly in contrast to the clearance of the dialyzer, remains unclear and has not been measured. Therefore, the primary research objective of this post hoc analysis of the Cyto-SOLVE study was to determine whether CS can adsorb lactate and can therefore contribute to a higher lactate clearance. The second research objective was to investigate whether its use leads to a change in lactate levels in patients’ blood and vasopressor dosage during application.

## 2. Methods

### 2.1. Study Setting

This was a post hoc analysis of the prospective Cyto-SOLVE study. The intention of the analysis was to best describe the adsorption spectrum of CS with regard to its potential removal of lactate. In the case of significant adsorption, routine use in lactic acidosis could contribute to faster pH normalization, whereas in the absence of adsorption, this indication would not justify its use. The local institutional review board approved the trial (registration number 21-0236). This study was registered at ClinicalTrials.gov (NCT04913298). Patients were included between May 2020 and May 2022 at two intensive care units (ICUs) during their stay at the LMU university hospital in Munich. Written informed consent was obtained from all patients or from their legal representatives. The indications for CS application were rhabdomyolysis (n = 20), acute liver dysfunction (n = 20), and hyperinflammation (n = 17).

### 2.2. Laboratory Measurements and Data Collection

Lactate was assessed using blood gas analysis (ABL800 FLEX, Radiometer GmbH, Krefeld, Germany) at the ICUs. For data evaluation, demographic data as well as clinical and laboratory variables were collected from the laboratory and patient data management system. Additionally, baseline characteristics such as age, gender, body mass index (BMI), 28-day mortality, Simplified Acute Physiology Score (SAPS) II, and the reason for ICU admission were evaluated on the treatment day. Lactate levels and the concentration of vasopressors were measured before and during the intervention.

### 2.3. Study Population

**Figure 1:**
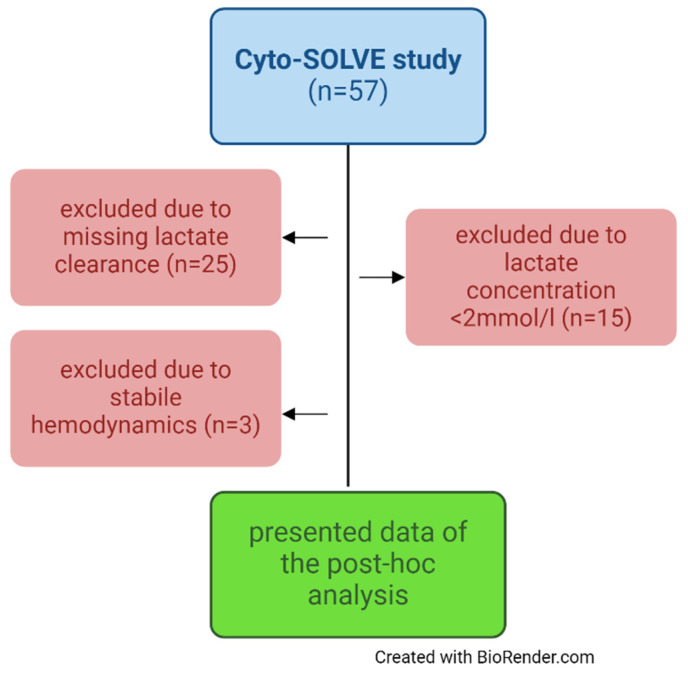
Flowchart of patient selection for post hoc analysis.

All patients from the Cyto-SOLVE trial were screened for inclusion in the analysis. Exclusion criteria included a lactate concentration < 2 mmol/L, no measurement of the lactate clearance, and no catecholamine requirement. All patients were treated with CKRT due to anuric or oliguric acute kidney injury, diagnosed by the KDIGO consensus criteria. CS was installed for the elimination of cytokines, myoglobin, or hepatotoxic substances in the CKRT device. CKRT was performed with the Fresenius MultiFiltrate circuit (MultiFiltrate Ultraflux^®^ AV 1000S, Fresenius Medical Care, Bad Homburg, Germany) either as continuous venovenous hemodialysis CVVHD (CiCa^®^, Fresenius Medical Care, Bad Homburg, Germany) or continuous venovenous hemodiafiltration CVVHDF post-dilution (MultiBic^®^, Fresenius Medical Care, Bad Homburg, Germany). CS was always installed post-dialyzer use for 12 h. Figure 1 shows the selection of the patients based on the exclusion criteria as a flow chart.

### 2.4. Blood Sampling

To evaluate the lactate adsorption and therefore the extracorporeal lactate clearance by CS, lactate was measured directly before and after the adsorber in the extracorporeal circuit five times during the application: 10 min after the initiation of CS (t1), as well as 1 (t2), 3 (t3), 6 (t4), and 12 h after initiation (t5). Arterial blood samples were taken shortly before initiation, as well as after 6 and 12 h (=end of therapy). The arterial lactate concentration was used as a surrogate for the concentration pre-dialyzer use. The clearance of CS as well as of the dialyzer at t1, t4, and t5 was calculated using the following formula:

$$Clearance\;\left(\frac{mL}{min}\right)=\left(bloodflow\right)\times \frac{concentration\;(pre-post)}{concentration\;(pre)}$$

### 2.5. Statistical Analysis

A post hoc power analysis was performed with G*Power version 3.1.9.7 to assess the significance of the data. In total, the lactate concentration was measured 65 times pre- and post-CS application (=total sample size). Assuming an alpha error of 5% and a relatively small effect size of 0.4 to detect even small effects from the application of CS, the present study yielded a power of 0.94. If no significant lactate elimination through CS was observed at the chosen effect size of 0.4, it could be assumed that the device does not directly contribute to increased lactate clearance. However, if a significant effect was observed, the results can help generate hypotheses for further studies.

Statistical analysis was performed with IBM SPSS statistics (Version 29.0. IBM Corp., Armonk, NY, USA). Continuous variables are presented as the median and interquartile range (IQR) or as the mean with standard deviation (SD). A *t*-test with dependent samples or, in the absence of a normal distribution, the Wilcoxon test, was performed to determine a significant change (*p* < 0.05) in the lactate concentration pre- and post-CS application, pre- and post-dialyzer use, and in patients’ blood, as well as in norepinephrine and vasopressin dosage during CS application. A correlation analysis was performed between the blood-flow and lactate clearance of both CS and the dialyzer. The lactate clearance was calculated using the above-mentioned formula.

### 2.6. AI Statement

To improve the linguistic quality of the manuscript, we used ChatGPT on January 9, 2025. Only linguistic revisions were made to the manuscript. No content-related adjustments were made with the help of AI (OpenAI (2025); ChatGPT (Version 4) [large language model], https://openai.com/chatgpt, accessed on 18 January 2025).

## 3. Results

### 3.1. Demographic and Clinical Data

The lactate concentration was measured 65 times pre- and post-CS application in a total of 14 patients during the application of CS. The indications for the initiation of CS were hyperinflammation and rhabdomyolysis, with 50% of patients in each group. The underlying diseases included intestinal ischemia (n = 2), acute respiratory distress syndrome (n = 2), resuscitation (n = 2), multi-organ failure (n = 2), compartment syndrome (n = 2), aortic dissection (n = 2), and medication toxicity (n = 2). The median age was 52 years, and 71% of the patients were male. All patients were critically ill, as reflected by a median SAPS II score of 67 points on the day of therapy. Detailed patient characteristics can be found in Table 1.

### 3.2. Lactate Concentration Pre-/Post-CS Application and Pre-/Post-Dialyzer Use

A total of 65 lactate pairs were measured pre- and post-CS application, and 37 pairs were measured pre- and post-dialyzer use, respectively. The mean lactate concentrations pre- and post-CS application were 6.7 (SD: 5.1) and 6.9 mmol/L (SD: 5.3), with no significant change when comparing pre- and post-CS lactate concentrations (RR: −0.2 mmol/L, 95% CI: −0.4–0.1, *p* = 0.13, Cohen’s d: 0.90). The mean lactate concentrations pre- and post-dialyzer use were 9.1 (SD: 6.3) and 7.1 mmol/L (SD: 5.5), with a significant (*p* < 0.01) reduction post-dialyzer use (RR: 2.1 mmol/L, 95% CI: 1.3–2.9).

### 3.3. Lactate Clearance of CS and the Dialyzer

**Figure 2:**
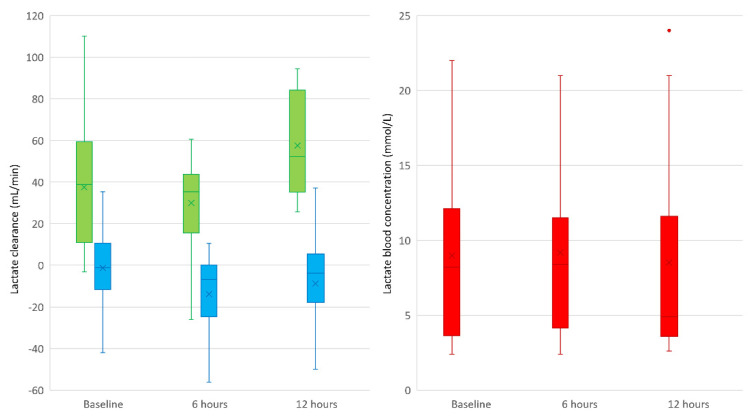
The lactate clearance of Cytosorb^®^ and the dialyzer and the lactate blood concentration. Note: the green boxplots illustrate a lactate clearance (mL/min) of about 30–40 mL/min for the dialyzer, which is comparable to the existing literature. The blue boxplots show the lactate clearance (mL/min) of Cytosorb^®^, which settles around 0 mL/min, with more than half of the calculated lactate clearances being negative. The red boxplots present the lactate concentrations at baseline and after 6 and 12 h in patients’ blood, with no significant change during the application of Cytosorb^®^ combined with continuous dialysis. The boxplot includes the interquartile range (IQR). The horizontal line represents the median, while the × represents the mean value. The whiskers are limited to 1.5 times the IQR, while outliers are shown as individual points.

The mean (SD) lactate clearance of CS and the dialyzer was −6 (21) mL/min and 39 (28) mL/min, respectively. There was no significant correlation between the blood flow and lactate clearance of CS or the dialyzer. In detail, the mean (SD) lactate clearances of CS at t1–t5 were −1 (17), −14 (19), −10 (23), 6 (18), and −10 (23) mL/min, respectively. The mean (SD) lactate clearances of the dialyzer at t1, t4 and t5 were 38 (29), 30 (23), and 57 (26) mL/min, respectively. The mean (SD) lactate concentrations in patients’ blood before the start, after six hours, and after twelve hours were 9.0 (5.9), 9.2 (5.8), and 8.5 (6.7) mmol/L, respectively, with no significant change. Figure 2 illustrates (A) the lactate clearance of CS and the dialyzer shortly after initiation, as well as 6 and 12 h after therapy, and (B) the arterial lactate concentration before initiation and after 6 and 12 h.

### 3.4. The Catecholamine Dosage Before, During, and at the End of CS Application

**Figure 3:**
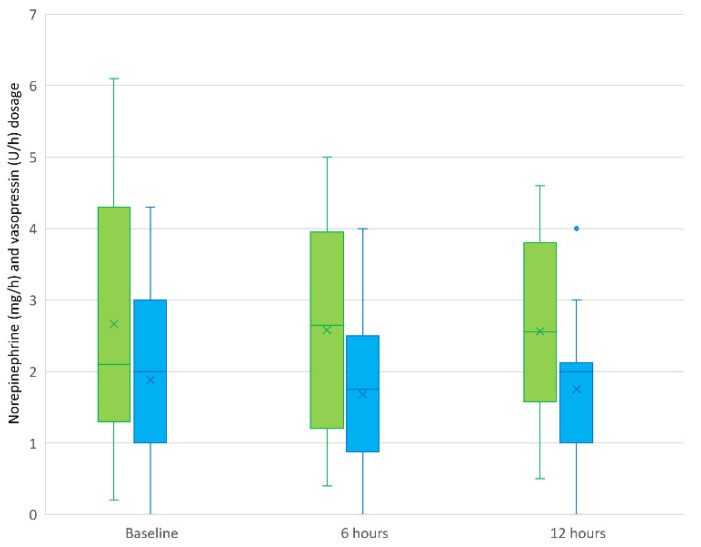
Norepinephrine and vasopressin dosage before, during, and at the end of Cytosorb^®^ application. Note: green boxplots illustrate norepinephrine dosage (mg/h), and blue boxplots show vasopressin dosage (U/h) at baseline and after 6 and 12 h. There was no significant change in catecholamine requirement during application of Cytosorb^®^. The boxplot includes the interquartile range (IQR). The horizontal line represents the median, while the × represents the mean value. The whiskers are limited to 1.5 times the IQR, while outliers are shown as individual points.

The norepinephrine and vasopressin dosages were determined shortly before initiation and after 6 and 12 h of treatment. The mean (SD) norepinephrine dosage at the different timepoints was 2.7 (1.7), 2.6 (1.4), and 2.6 (1.2) mg/h, respectively. There was no significant change in norepinephrine requirement at any of the periods. The mean (SD) vasopressin dosage at different timepoints was 1.9 (1.1), 1.7 (1.1), and 1.8 (1.0) U/h, respectively. There was also no significant change in the vasopressin dosage at any of the periods. Figure 3 illustrates the course of norepinephrine and vasopressin dosage before, during, and at the end of CS application.

## 4. Discussion

This post hoc analysis of the Cyto-SOLVE trial demonstrates that CS cannot directly adsorb lactate in critically ill patients who are simultaneously treated with CKRT, and that it does not lead to an increased extracorporeal elimination through its application. In the post hoc power analysis, a relatively small effect size of 0.4 was chosen. The aim was to detect even a potentially small to moderate effect of CS on lactate clearance; to justify a prospective study, the results should be positive. However, we observed no significant change in the lactate concentration when comparing the pre- and post-CS levels in the extracorporeal circuit, whereby the significance of the results is underlined by a power of >90% with 65 measured lactate concentrations at an alpha level of 5%.

The adsorber CS has been approved for several years and is used in a variety of applications. The primary intention was the adsorption of pro-inflammatory cytokines in sepsis to achieve faster hemodynamic stabilization.

In addition to the removal of cytokines, some publications describe that CS can also improve lactate clearance by extracorporeal lactate elimination. Specifically, two prospective trials reported a decrease in lactate levels in patients with septic shock treated with CS, although they lacked a control group [19,22]. Similar results were described in two retrospective analyses, with a significant decrease in lactate concentrations [18,21]. The problem is that these studies lack a control group, and both causal therapies (e.g., antibiotics in sepsis and dialysis in lactic acidosis) as well as adjunctive treatments (e.g., fluid substitution) affect or reduce lactate concentrations. Adequate fluid administration leads to a reduction in lactate levels, while newer data suggest that liberal fluid administration is not superior to a more restrictive approach [23,24]. Furthermore, using CKRT leads to an increased lactate clearance of about 40 mL/min depending on the blood flow of the device [25,26,27]. These data are comparable with our data, which underscores the validity of our data.

Focusing on studies with a control group, three matched datasets showed that lactate blood concentrations in critically ill patients do not differ with or without the use of CS [28,29,30]. However, none of these studies have measured lactate levels directly pre- and post-CS application to evaluate its direct effect. Therefore, our data are first in line with the abovementioned studies with a control group and significantly contribute to the understanding of the device. Its use to increase extracorporeal lactate clearance does not appear to be justified by our data.

Surprisingly, we observed a negative lactate clearance by CS in 54% of the measurements. Recently, Buhlmann et al. reported the desorption of various substances, particularly after several hours of application [31]. This can also apply to lactate, although this phenomenon has only been described for substances that are normally adsorbed by CS, like bilirubin. Another possible explanation is that lactate might be generated within the adsorber, for example, through platelets that remain in the adsorber, as recently described by *Brozat* and colleagues [32].

Knowledge of the exact adsorption spectrum of non-selective adsorption methods is fundamentally important to ensure their rational use with known side effects. Previous studies, for example, have shown that antibiotics can be adsorbed through their use [33,34], whereas there is no evidence of ammonia adsorption despite this being previously described [35]. Since the adsorber primarily eliminates molecules larger than 5 kDa [16], lactate might be too small to bind to the beads.

The secondary research objective was determining whether the use of CS changes the catecholamine requirement during its application. All patients in this analysis received catecholamine therapy, with most being treated with both norepinephrine and vasopressin, showing no significant change during the application of CS (Figure 3). This finding is consistent with the results from other prospective studies that included a control group.

For example, Monard et al. found no significant difference in the norepinephrine dosage after treatment with CS between critically ill patients receiving CS and those in a control group [36]. Additionally, shock reversal was significantly less pronounced in the CS group after 24 h compared to the control group. Regarding lactate levels, patients without CS had significantly higher concentrations before the study (7 vs. 4 mmol/L), with a more pronounced drop after 24 h compared to patients with CS treatment (1 vs. 2 mmol/L). Similar findings were reported in a multicenter randomized controlled trial involving patients with COVID-19 and vasoplegic shock and a matched controlled study, where no differences were observed in the catecholamine requirements or lactate concentrations between the groups [29,37].

To summarize, the adsorber CS cannot adsorb lactate from the blood in patients with hyperlactatemia. This finding is new and has not been previously described, thus contributing to a better understanding of the device. In half of the cases, even higher lactate concentrations were observed after the adsorber’s application. This should be further investigated, as an additional lactate load due to the use of CS, which is typically applied in severely ill patients, could be associated with harmful side effects. In contrast, the dialyzer demonstrated a lactate clearance comparable to that in the literature. The lack of hemodynamic stabilization has already been observed in several studies with control groups, and the ongoing multicenter PROCYSS study (ClinicalTrials NCT04963920), which focuses on this issue, will hopefully provide new insights in the future.

Finally, this study has several limitations. First, it was a post hoc analysis limited to 65 lactate measurements pre- and post-adsorber application. As no control group without CS treatment is available, statements regarding the change in lactate blood concentrations should be assessed with caution as many other therapies, such as fluid administration and antibiotic therapy, also have a direct impact. However, the primary research question of whether CS can adsorb lactate can validly be answered. Second, due to the lack of a control group, evaluating clinical progression without treatment with CS was not possible, especially focusing on the catecholamine requirement. For this purpose, the data were compared with those in the existing literature. Third, the study population was treated with multiple interventions (i.e., antibiotics, vasopressors, dialysis, surgical intervention, and ECMO) for different disease patterns. The effect of the individual interventions on the lactate concentration in the blood and catecholamine requirement is unclear. Lastly, patients were only treated with one adsorber. It remains unclear whether a more frequent application of CS might result in a greater benefit for the patient. To enhance the power of future randomized studies, patients should be stratified based on the extracorporeal techniques used, and additional therapies should be standardized as much as possible. In this way, the actual effect of CS can be distinguished from that of other treatment methods in the future.

## 5. Conclusions

The adsorber CS cannot directly adsorb lactate in patients with hyperlactatemia and therefore cannot directly improve lactate clearance. In fact, even higher lactate levels were measured after the adsorber’s application than before, suggesting lactate desorption or production within the adsorber. Therefore, it is not suitable for achieving faster extracorporeal lactate elimination. This new information should be considered when using CS in clinical practice. It is desirable for future studies to further investigate the potential desorption of lactate by CS.

## Figures and Tables

**Table 1:** Detailed patient characteristics.

	n (%) or Median [IQR]
Patient Characteristics	
Age (years)	52 [45, 63]
BMI (kg/m^2^)	23.4 [21.3, 26.3]
28-day mortality	12 (85.7)
SAPS II score on treatment day	67 [60, 77]
Dialysis characteristics	
Dialyzer	Multifiltrate, Fresenius Ultraflux AV 1000 S, surface area: 1.4 m^2^, elimination spectrum: 0–15 kDa
CVVHD (CiCa^®^)/CVVHDF (MultiBic^®^)	6 (43)/8 (57)
Blood flow (mL/min)	200 [100, 260]
Net ultrafiltration (mL/h)	0 [0, 0]
Fluid balance (mL/24 h)	6550 [2975, 9120]
Laboratory parameters	
Interleukin-6 (pg/mL) before CS	7416 [868, 23,459]
Interleukin-6 (pg/mL) after CS	5512 [304, 22,786]
Myoglobin (ng/mL) before CS	40,480 [11,989, 81,947]
Myoglobin (ng/mL) after CS	42,923 [9108, 86,747]
Bilirubin (mg/dL) before CS	3.2 [2.0, 9.0]
Bilirubin (mg/dL) after CS	2.8 [2.0, 6.1]

## Data Availability

The datasets generated and/or analyzed during the present study are not publicly available, but they are available from the corresponding author upon reasonable request.

## Abbreviations

AKI	acute kidney injury
BMI	body mass index
ECMO	extracorporeal membrane oxygenation
CKRT	continuous kidney replacement therapy
CS	Cytosorb^®^
CVVHD	continuous venovenous hemodialysis
CVVHDF	continuous venovenous hemodiafiltration
ICU	intensive care unit
IQR	interquartile range
SAPS II	Simplified Acute Physiology Score II
SD	standard deviation
